# The cost of saving: How photos and screenshots impair memory

**DOI:** 10.3758/s13421-025-01711-2

**Published:** 2025-04-07

**Authors:** Rebecca Lurie, Sophia P. Fabrizio, Deanne L. Westerman

**Affiliations:** Department of Psychology, Binghamton University, State University of New York, PO Box 6000, Binghamton, NY 13902-6000 USA

**Keywords:** Memory, Cognitive offloading, Technology, Photographs

## Abstract

The photo impairment effect refers to worse memory for experiences that are photographed compared with those that are not. One proposed explanation for this effect is that photo-taking divides attention between the event and the actions required for photography. However, the results of the present study challenge this account. Specifically, we found that the magnitude of the photo impairment effect did not increase with task complexity, undermining the idea that divided attention is the primary cause. Across three experiments, participants viewed art presented on a computer or their own smartphones and either photographed or took screenshots of the images. Memory was consistently worse for images saved by any of these methods. Notably, screenshotting had particularly detrimental effects on memory, despite being the least complex saving method. Furthermore, the impairment did not vary based on whether participants used a familiar device (their own smartphone) or an unfamiliar, experimenter-provided camera. These findings suggest that divided attention alone cannot account for the photo impairment effect.

## Introduction

The rapid advancement of technology has sparked widespread concern about its potential negative effects on our basic cognitive processes, particularly its impact on learning and memory (Carr, 2008; Hari, 2022; Hipp et al., 2020; Marche, 2002; Pasquinelli, 2018; Seal, 2022; Wilmer et al., 2017). While recent discussions have focused on the recent development of Generative AI (artificial intelligence) tools (Huang, 2023; Kirschenbaum, 2023; Roose, 2023), the idea that technological advances that enable us to store, process, and retrieve information might diminish our own mental faculties is longstanding. In Plato’s dialogue *Phaedrus*, Socrates warned that writing as a means of communication would weaken memory and provide only the “appearance of wisdom, not true wisdom” (*Phaedrus* 275a) (Plato 1995). Similar fears were expressed following the invention of the printing press, which made it possible to externalize knowledge storage (Eisenstein, 1979). As technology increasingly mediates how we experience, store, and access information, questions about its influence on our mental faculties have again come to the forefront.

Research on this topic suggests that the concerns are not entirely unfounded, particularly with regard to memory, as reliance on technology to save information has been found to negatively affect our ability to remember it. This was first demonstrated by Sparrow et al. (2011; see also Storm & Stone, 2015), who found that participants remembered information more poorly when they believed that it was saved by a computer, a finding that is sometimes called *The Google Effect* or *digital amnesia* (Kapersky Lab, 2015).

If memory is impaired when information and events are stored externally, then one might wonder about the impact of the widespread use of smartphones, which allow us to easily memorialize nearly every facet of our lives. Similarly, other types of information that are accessed from the devices can easily be saved with screenshots and screen recordings. In what way is our memory for these experiences and information impacted when they are captured on an external device?

A number of studies have investigated the memorial fate of photographed information, both in controlled laboratory settings (Lurie & Westerman, 2021; Soares & Storm, 2018) and in more naturalistic environments such as museums (Barasch et al., 2017; Henkel, 2014) and bus tours (Zauberman et al., 2015). Although one study found that photographed events are remembered better than non-photographed events (Barasch et al., 2017), the balance of results show a consistent pattern: Events and objects are remembered more poorly if they are photographed than if they are just experienced without being photographed (Henkel, 2014; Lurie & Westerman, 2021; Niforatos et al., 2017; Soares & Storm, 2018). This pattern of results has been termed the “Photo-taking Impairment Effect” (Henkel, 2014).

## Why does photo-taking impair memory?

Several explanations of the photo-impairment effect have been proposed. One intuitive explanation is that photo-taking divides a person’s attention during the experience (Henkel, 2014). That is, our limited attentional resources are split between the act of taking the photo and the in-the-moment processing of the object or event that we wish to capture, leading to poorer memory for the event (e.g., Anderson & Craik, 1974; Baddeley et al., 1984; Craik et al., 1996). A second explanation is based on the notion of cognitive offloading, which refers to the use of some physical action (e.g., writing something down or taking a photograph) to reduce the cognitive demand of a task (Risko & Gilbert, 2016). An offloading account posits that photo-takers rely on the device to “remember” the information for them, leading to worse memory for photographed information, compared with relying solely on their own memories for non-photographed information. Although the precise cognitive mechanisms underlying offloading are not clear, such an account generally suggests that fewer processing resources (either at initial encoding or during the retention interval) would be devoted to the photographed information. According to this explanation, information contained in a screenshot or a screen recording may be similarly poorly remembered, as the person would rely on their device to remember the information contained in the screenshot. A third explanation, termed attentional disengagement (Soares & Storm, 2018), suggests that attention is generally reduced, rather than divided, when photographing an event.

The divided attention and attentional disengagement accounts are similar in so far as they are based on reduced attentional resources devoted to the event that is being photographed. However, each of these candidate mechanisms makes predictions that are dissociable. A divided attention explanation suggests that simultaneously performing a secondary task impairs encoding and subsequent memory of material, and that the magnitude of the impairment should be proportional to the difficulty or complexity of the secondary task (as a more complex task is more demanding of attentional resources). Therefore, if divided attention is a major contributing factor to the photo impairment effect, then manipulating the type of photo-taking task to vary its complexity, and thus the degree to which the task draws attention away from the art itself, should modulate the impairment. On the other hand, the attentional-disengagement hypothesis argues that when taking photos, participants become broadly disengaged from the experience and in turn fail to properly encode the material. Soares and Storm (2018) proposed this account after finding the photo impairment effect even when participants were given extra time to encode after taking the photographs (which one would expect to overcome any effects of divided attention). In the same study, they also found that the effect persisted even when the photos were immediately deleted (which contradicted a conscious cognitive offloading, which requires the saving method to be reliable). Soares and Storm (2018) suggested that their results could reflect a “metacognitive illusion,” where the participants disengage from the experience because they believe the material has been encoded already, and may not utilize strategies to support encoding and consolidation even when given additional time after taking the photo. They also speculate that our learned association between photo-taking and the ability to offload the information could lead to an unconscious, automatic disengagement despite an unreliable saving method. This conceptualization of the attentional-disengagement account suggests that the photo impairment effect would still be present and unaffected by the complexity of the photo-taking task.

It is also important to consider that the candidate mechanisms for the photo-impairment effect may not be entirely mutually exclusive. For instance, a participant, realizing that the camera can be used to “remember” the information (offloading), may disengage from the experience (attentional disengagement) or allocate their attentional resources toward framing the shot and operating the camera.

The current study has two goals that are focused on the predictions of the divided attention account. The first goal is to determine whether the impairment for photographed information extends to other saving tasks, such as screenshots. The second goal is to determine whether the magnitude of the photo-impairment effect is modulated by the complexity of the “saving” task. As the negative effects of divided attention on memory should depend on the complexity/difficulty of the secondary tasks, the photo impairment effect should be larger if the “capturing” task is more attention demanding and vice versa (McDowd, 1986; Oberauer & Kliegl, 2004). On the other hand, offloading and attentional disengagement would be less likely to be impacted by the complexity of the photo taking task.

## Power analysis

A power analysis was conducted using G*Power (Faul et al., 2007), with a power level of 0.9 and an alpha level of 0.05. The power analysis was conducted using a medium effect size (*d* = 0.5) and a correlation among repeated measures of 0.5 was used. The medium effect size was a conservative choice, as a study using similar procedures and stimuli by Lurie and Westerman (2021, Experiment 1) found a large photo impairment effect (*d* = 1.25). The medium effect size was chosen to allow for a smaller effect than the simple photo impairment effect, as might be expected when comparing two different capturing methods (e.g., own camera vs. experimenter-provided camera). The power analysis revealed that an *N* of 36 was needed for Studies 1, 2, and 3 to detect an effect of this magnitude. We attempted to test approximately that number in Experiments 1–3, with some deviations due to term-related variations in the number of participants available (e.g., we fell slightly short of that goal in Experiment 3 due to difficulty recruiting undergraduate student participants during that particular point of the semester). Experiment 4 was more exploratory and used a large *N* for reasons discussed in the methods section of that experiment.

## Study 1

Study 1 attempted to address a divided attention account of the photo impairment effect by testing the impact of device familiarity. The procedures of Experiment 1–3 were closely modeled on a study by Lurie and Westerman (2021), which found a robust photo impairment effect on a forced-choice recognition test that presented the target amid two visually similar lures. In the present experiment, incidental encoding procedures were used for consistency with past research (e.g., Lurie & Westerman, 2021) and to mimic real-world conditions (as people probably do not encode experiences with the expectation of an imminent memory test).

In Study 1, participants used a tablet provided by the experimenter or they used their own smartphone to take photos of the art. If the photo-taking impairment is due to participants dividing their attention between the photo-taking task and the viewing task, then we would expect the impairment to depend on device familiarity. Specifically, participants should show worse memory when using the unfamiliar device provided by the experimenter than when using their own phone, as practice with a secondary task has been shown to reduce the impact of divided attention during encoding (McDowd, 1986; Oberauer & Kliegl, 2004). On the other hand, a cognitive offloading and/or attentional disengagement account would not predict differences depending on device familiarity.

### Method

#### Participants

Participants included 38 undergraduate students who participated in exchange for credit toward a course requirement. This study was approved by the university’s institutional review board (IRB). Demographic information was not collected for Studies 1–3. However, participants were drawn from the same pool as the participants in Study 4, for which demographic information is available.

#### Materials

One hundred and thirty-five images depicting pieces of visual art were used as stimuli. Forty-five were used for the study phase, and 90 were used as foils on the forced-choice recognition test. A variety of artwork was used, including paintings, sketches, and photographs. The foils were images of art that were perceptually and conceptually similar to the study items and were composed of two additional pieces from the same artist or two pieces by a different artist that depicted the same object. Abstract art and familiar artworks were avoided. The images were obtained from the Artstor Digital Library (http://library.artstor.org), and most of the images were used by Lurie and Westerman (2021). We note that in the Lurie and Westerman study, the use of perceptually-similar lures were critical to the hypotheses of that study, and we maintained the stimuli and general procedures because they produced robust results and accuracy that is not near the ceiling, as can happen when images are used as stimuli (e.g., Brady et al., 2008). An example study and test trial can be found on the Open Science Framework (OSF) page for this study. Items were presented to participants using PsychoPy (Peirce, 2007) and Pavlovia (pavlovia.org).

#### Procedure

Participants were tested in person and completed the experiment individually. They were not told that they were in a memory experiment. Rather, they were informed that we were interested in how they experience art. Participants were seated approximately 2.5 feet away from an 18-in. computer monitor. Each image was displayed in the center on the computer screen, one at a time, and participants viewed and engaged with the art at their own pace. The participants were shown three blocks of 15 items and were asked to perform a different task during each block. For one block, they were asked to use an experimenter-provided Android tablet to take a photo of each piece of art before moving on to the next one. The tablet measured 10.75 × 6.5 in. with a screen size of approximately 9 × 5 in. For a second block, participants were asked to take a photo using their own smartphone. For a third block, participants were asked to view the items. Participants proceeded through each block at their own pace, pressing the spacebar when they were ready to move on to the next piece of art. When participants photographed the art, a small depiction of the previous picture was temporarily visible in a bottom corner, as is typical on smartphones and tablets; however, this image was small, and it was not possible to discern any details from the image. During the camera block, on-screen reminders to take a photograph appeared 1 s after the artwork was displayed and stayed on-screen until the participant progressed to the next piece.

The order of the blocks was counterbalanced among participants, and the items that appeared in each block were randomized for each participant. Prior to the study phase, participants completed a practice trial in which the experimenter showed the participant which button to press on the provided device to take their photo, and participants took a photo of the practice item.

Upon completion of the study phase, participants completed a distractor task that involved coloring a mandala for 20 min. This particular distractor task was selected to feed into the impression that the study was about a person’s experience with art. After the distractor task, participants were tested on the artwork. The test consisted of a three-alternative forced-choice recognition test. There were 45 test trials. In each, an image from the first phase was presented alongside two visually similar lures. The order of the test items was randomized, as well as the location of the target and lures on the screen. Participants were asked to indicate which image they had seen during the study phase by pressing the number key (1, 2, or 3) that corresponded to the target location.

### Results

There were no main effects nor interactions related to block order, therefore the data were collapsed across study block. A repeated-measures ANOVA (photo condition: experimenter camera vs. own camera vs. no photo) was conducted on the percentage of correct responses. There was a main effect of photo condition, *F*(2,37) = 9.30, *p* < 0.001, *MSE* = 258.23, η^2^p = 0.20. Taking photos with the provided camera (*M* = 64.91%, *SD* = 17.45) led to worse memory than simply viewing the art (*M* = 77.72%, *SD* = 17.76), *t*(37) = 3.65, *p* < 0.001, *SE*_*diff*_ = 3.51, *d* = 0.59, 95% CI of *d* = [0.24, 0.93]. Taking photos with their own phone (*M* = 63.16%, *SD* = 24.00) also led to worse memory than viewing, *t*(37) = 3.40, *p* = 0.002, *SE*_*diff*_ = 4.28, *d* = 0.55, 95% CI of *d* = [0.21, 0.89], but there was no difference in memory between the two types of photos, *t*(37) = 0.55, *p* = 0.58, *SE*_*diff*_ = 3.19, *d* = 0.09, 95% CI of *d* = [−0.22, 0.41]. A supplementary Bayesian analysis supported the interpretation of no difference in accuracy for photos taken with a participant’s own camera and an experimenter-provided camera, BF_01_ = 4.97. The result indicates the data are approximately 4.97 times more likely under the null hypothesis than the alternative hypothesis, offering moderate support for the null hypothesis.

As noted in the procedures, participants proceeded through the encoding and tests phases at their own paces, and we made no attempt to equate the amount of time participants spent on each trial. We did record the amount of time that participants spent on each trial. As might be expected, there were differences in the amount of time spent on each trial depending on encoding conditions, *F*(2, 37) = 8.16, *p* < 0.001, η^2^p = 0.13. The mean trial time was 7.54 s (*SD* = 1.68 s) per trial when they had to photograph the image with the unfamiliar experimenter provided tablet, 3.87 s (*SD* = 2.38 s) when they had to take a photo with their own camera and 4.86 s (*SD* = 6.48 s) when they simply viewed the photo. A Tukey’s post hoc test indicated that the experiment-provider camera condition produced longer trials times than when they used their own camera (*p* < 0.001), and when they viewed the art (*p* = 0.014). The difference between photographing with their own phone and viewing was not significant, *p* = 0.542. The important thing to note from this analysis is that the total amount of time spent on each trial does not correspond to recognition accuracy. This result dovetails with the findings of Soares and Storm (2018, Experiment 2), who also found that trial time had no bearing on the photo-impairment effect.

## Study 2

Study 1 showed no difference in memory between a familiar device (a participant’s own smartphone) and an unfamiliar device (an experimenter-provided tablet), consistent with an offloading and/or attentional disengagement account. The goal of Study 2 was to extend this finding to task complexity. In Study 2, two different types of tasks were used to capture the images presented as stimuli: photo-taking with an unfamiliar device (more complex) and screenshotting (less complex). If divided attention is a better account of the photo-taking impairment effect, then the more complex and attention demanding photo-taking task (which involved lifting the tablet, pointing the camera, and taking a photo) should lead to greater impairment than the less complex task (pressing keys on the keyboard to take a screenshot). On the other hand, if the memory impairment is a product of offloading and/or attentional disengagement, the complexity of the capturing task would seem less likely to impact the impairment.

### Method

#### Participants

Participants included 42 undergraduate students who participated in exchange for partial course credit.

#### Materials

Study 2 used the same materials as Study 1.

#### Procedure

Participants were shown three sets of art, as in Study 1. For one set, they were asked to use an experimenter-provided Android tablet to take a photo of each piece of art before moving on to the next one. Study 2 used the same computers and tablet as Study 1, and participants were shown a brief demonstration of how to use the camera before starting the experiment. For another block, they were asked to press a combination of three different keys to take a screenshot of each piece of art before moving on to the next. For the last block, they were asked to simply view the items. The order of these blocks was counterbalanced among participants, and the items that appeared in each block were randomized for each participant.

Participants completed two practice trials prior to viewing the art. In the first practice trial, they were told which keys to press to take their screenshot. The keys were marked with colored stickers to ensure that participants would be able to quickly identify the keys to press when they needed to take screenshots. Unlike the photographing condition, no miniature version of the image was visible after the screenshot was taken. After taking their practice screenshot, they were shown where on the computer the screenshot had been saved. That folder remained visible in the bottom taskbar of the computer for the duration of the experiment. For the second practice trial, participants were given the tablet that would be used to take their photos, and they were shown which button to press within the tablet’s camera application to take their photos. After completing the camera practice trial, the experimenter took the tablet from the participant to hold onto during the *view* and *screenshot* blocks.

The distractor task and test were identical to Study 1.

### Results

**Fig. 1:**
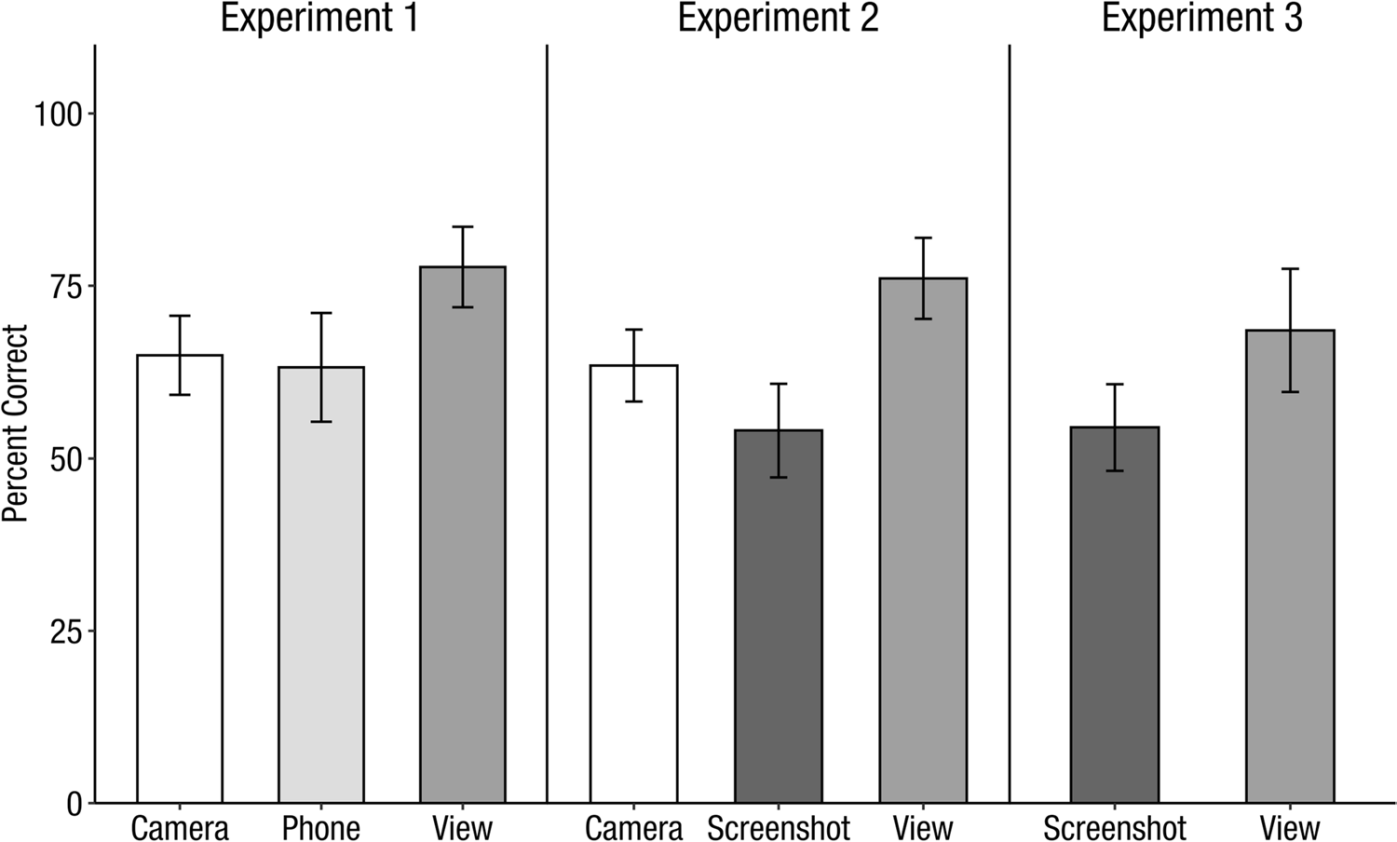
Recognition memory performance Studies 1–3. Error bars represent 95% confidence intervals

Three participants were excluded from analysis because they had difficulty reliably taking screenshots with the computer. There were no main effects nor interactions related to block order, therefore the data were collapsed across study block. A repeated-measures ANOVA (photo condition: physical camera vs. screenshot vs. no photo) was conducted on the percentage of correct responses on the three-option forced-choice recognition test. The recognition results are summarized in Fig. 1. There was a main effect of photo condition, *F*(2,38) = 25.22, *p* < 0.001, *MSE* = 189.34, η^2^p = 0.40. Taking photos (*M* = 63.42%, *SD* = 16.10) led to worse memory than simply viewing the art (*M* = 76.07%, *SD* = 18.14), *t*(38) = 4.74, *p* < 0.001, *SE*_*diff*_ = 2.67, *d* = 0.76, 95% CI of *d* = [0.40, 1.11]. Taking screenshots (*M* = 54.02%, *SD* = 20.85) also led to worse memory than both viewing, *t*(38) = 6.27, *p* < 0.001, *SE*_*diff*_ = 3.52, *d* = 1.00, 95% CI of *d* = [0.61, 1.39], and taking photos, *t*(38) = 3.03, *p* = 0.004, *SE*_*diff*_ = 3.10, *d* = 0.49, 95% CI of *d* = [0.15, 0.81].

The amount of time that participants spent on each trial was analyzed with a one-way repeated-measures ANOVA. As might be expected, there were differences in the amount of time spent on each trial depending on the encoding condition, *F*(2, 38) = 38.02, *p* < 0.001, η^2^p = 0.42. The mean trial time was 6.86 s (*SD* = 1.74 s) per trial when they had to photograph the image with the experimenter provided tablet, 3.48 s (*SD* = 1.83 s) when they had to take a screenshot, and 3.30 s (*SD* = 2.17 s) when they only viewed the art. A Tukey’s post hoc test indicated that the camera condition was significantly longer than the screenshot and view-only conditions (*p*s < 0.001), but that the screenshot and view conditions were not significantly different from each other (*p* = 0.92).

The results showed the usual photo-taking impairment effect and demonstrated that screenshotting produced an even larger deficit than photographing. Because the screenshotting task should be less complex, this result is evidence against a divided attention account of the photo impairment effect. However, a divided attention account may still be viable for two reasons. First, screenshotting involved pressing a set of keys simultaneously. It is possible that participants may have been looking at these keys on some of the trials, rather than looking at the art, leading to poorer encoding. Second, taking a screenshot may have been a more complex secondary task than we realized. Participants were using an unfamiliar keyboard to perform the task, and perhaps that was more attentionally demanding than using an unfamiliar tablet. We made several methodological changes in Study 3 to address these concerns.

## Study 3

The goal of Study 3 was to replicate the photo impairment effect with screenshots using a more familiar method of taking the screenshot. Participants used their own smartphones to view and screenshot art that was presented during the encoding phase. Because they viewed the art on their own device, we did not include a photo-taking condition (as asking participants to take a photo of their phone screen with a tablet camera seemed strange and impractical). In this context, an offloading or attentional disengagement account would predict that memory will be poorer when screenshots are taken, as participants would still be relying on the device to remember the information for them and/or they might still disengage from the experience of viewing art. Conversely, a divided attention account would predict that the impairment would be minimal when taking screenshots with their own device, as task familiarity and practice has been shown to reduce dual-task costs (McDowd, 1986; Oberauer & Kliegl, 2004). Indeed, in Experiment 2, trial times were comparable for the screenshotting and viewing conditions, suggesting that the task demands of screenshotting are pretty minimal.

### Method

#### Participants

Participants included 31 undergraduate students who participated in exchange for partial course credit.

#### Materials

Study 3 used only the original 32 study items and 64 foils from Lurie and Westerman (2021), Experiment 1.

#### Procedure

Participants completed the study phase on their own device. The recruitment materials informed participants that the experiment was interested in their experience of art. They were asked to bring their phones, and familiarity with taking screenshots on their smartphone was a condition for inclusion in the study. They were also told that their device should have enough battery to last 5–10 min and enough storage to save about 20 images. When they arrived at the lab, all of the participants indicated familiarity with the process of taking screenshots on their phones. To begin the experiment, participants used their phone to scan a Quick Response (QR) code that took them to a Pavlovia (pavlovia.org) link. All instructions and artwork were displayed on the participant’s phone. After completing a practice screenshot to confirm their familiarity with the mechanism on their device, participants were shown two sets of art, presented in blocks of 16 images. For one set, they were instructed to take a screenshot of each piece of art. For the other set, they were told to simply view each piece. When the images were screenshotted, the screen briefly flashed white and then a small version of the image appeared in the bottom left corner briefly. The order of these blocks was counterbalanced among participants, and the items that appeared in each block were randomized for each participant. The distractor task and the test were identical to Study 1 (i.e., participants took the test on a computer, not on their phone), but included only the 32 items (with two lures for each target) that corresponded to items used in the study phase. The order of the test items and target/lure location were randomized.

### Results

Two participants were excluded because of a computer error in which their test responses were not recorded, and one participant was excluded because their device was too old to properly display the study phase items. All but one participant used iPhones to complete the study phase. There were no main effects nor interactions related to block order, therefore the data were collapsed across study block. A paired-samples *t*-test was used to compare accuracy between art that had been screenshotted and art that had been viewed. Memory was worse for items that were screenshotted (*M* = 54.46%, *SD* = 16.12) than items that were viewed (*M* = 68.53%, *SD* = 23.04), *t*(27) = 3.04, *p* = 0.005, *SE*_*diff*_ = 4.62, *d* = 0.58, 95% CI of *d* = [0.17, 0.97].

We could not analyze the timing of the study trials in this experiment, as our program was not able to record the timing of images presented on the participants’ phones.

## Study 4

In Study 2, images that were saved (via screenshot or photograph) showed a profound memory deficit compared with images that were viewed. The impairment to memory was significantly greater in the screenshotting condition, which is contrary to a divided attention account. In fact, the larger impairment for images saved via screenshot also suggests that more offloading and/or disengagement may occur when screenshotting versus photographing. Study 4 was an attempt to explore the plausibility of the idea that screenshotting is more likely to be associated with cognitive offloading by examining the goals associated with screenshotting versus photographing.

Soares and Storm (2022) administered a survey to two samples of undergraduate students to explore their goals for taking photos. To complete the survey, participants accessed the last six photos in their phone’s photo album and reported their goal when taking each photo. The open-ended responses were then classified as belonging to different goal-types (e.g., to create a memento, to offload, to fulfill a social goal, and so forth). Particularly relevant to the current study is the finding that offloading was the intended goal of 12% and 15% (in samples 1 and 2, respectively) of the photos taken. Their survey did not consider the goals for screenshotting, so we adapted their survey to include both screenshotting and photographing to explore the idea that screenshotting may be an activity more closely associated with offloading compared with photographing.

### Method

#### Participants

Participants included 308 undergraduate students (190 females and 111 males) with an average age of 18.93 years (*SD* = 0.99) who participated in exchange for partial course credit. Subjects were recruited in large numbers to offer credit opportunities to students who needed to complete their course requirement toward the end of the semester.

#### Materials

We developed a 28-item Qualtrics (qualtrics.com) survey based on methods used by Soares and Storm (2022). The key differences in our adaptation were that our survey included questions about screenshots, and we gathered multiple-choice (vs. open-ended) responses. Participants were asked to consider their six most recent photographs and screenshots taken on their cell phones. For each of the 12 images, they selected what they believed to be the primary goal for saving that image. Each of the five goals was paired with a brief description to provide clarity, and an “Other/I don’t remember” option was included for those who could not recall their goal or find that none of these goals fit their recollection. The descriptions provided to participants were based on those in the coding manual for Soares and Storm (2022), which the authors supplied to help us develop our survey (https://osf.io/z2sk4/). The memento goal was described as “to later cue your memory for the event/information when looking back; to save the moment.” Offloading was selected if “the phone can hold onto information so you do not need to remember it yourself; to store information for future reference; memory replacement” accurately depicted their goal. Participants were to select the aesthetic goal when the intention was “to capture or appreciate the beauty in something.” Self-representation goals indicated that the image was meant to “represent or otherwise express yourself (e.g., to track your progress/change over time; because you felt confident, etc.).” An image with a social goal was captured “to develop an identity or online persona, to communicate information to others (to post online, to sell something online, to send to someone).” Participants were also asked “Where is the information needed to accomplish your goal stored?” based on a question included in Study 2 of Soares and Storm (2022). Their response was a rating on a Likert-type scale that ranged from 1 (*entirely in the photo*) to 7 (*entirely in my organic memory*). This was included for completeness but was not central to our investigation (however, the data are available at the OSF link for this study). The survey ended with demographic questions regarding age, gender, and race/ethnicity.

#### Procedure

Participants were recruited via an online experiment-scheduling platform. The recruitment materials included the request that the survey be completed on a laptop or computer and that they have their smartphone available for reference. After signing up, participants were redirected to a Qualtrics link where they read a consent form before completing the survey. Subjects were informed that they would be answering questions regarding the most recent photographs and screenshots on their phone that they had personally and intentionally taken. Multiple attempts at taking a photo of something or duplicates would only count as one image, and the content of the images would not be questioned.

The first block of questions involved the last six photographs they had taken, starting with the most recent and moving backward. For each image they selected the goal that best fit their reason for taking the photo, as well as the rating of where the information to meet that goal was located. They were reminded each time that screenshots did not count as one of their photographs for this section. The second block of questions were nearly identical, instead asking about the six most recent screenshots they had taken. Lastly, they completed a few demographic questions.

### Results

Two subjects were dropped from the analysis due to a lack of compliance with survey instructions, leaving data from 306 participants to be analyzed. If one or two of the 12 primary questions were left blank, the answers were re-coded as “Other/I don’t know” as the goal for that image. A total of four responses across three participants were re-coded. The dependent variable was the number of photos/screenshots that fell into each of the six response categories. The percentages of each image type attributed to the different goals are displayed in Table 1. The results showed different patterns for the goals of screenshots and photos. The key reason for doing this survey was to see if offloading was more likely to be a reason to take a screenshot versus a photo. We found that to be the case; the goal of offloading was more common with screenshots than with photos, *t*(305) = 7.24, *p* < 0.001, *SE*_*diff*_ = 0.09, *d* = 0.41, 95% CI of *d* = [0.30, 0.53]. Although we did not have hypotheses about the distribution of the response categories, the photo versus screenshot comparisons for all goals were significantly different (with Bonferroni correction), *p*s < 0.001.

**Table 1:** Percentage of goals for photographs and screenshots

Image type	Offloading	Memento	Social	Self	Aesthetic	Other	Total
Photograph	19.23	31.05	20.26	8.93	16.34	4.19	100
Screenshot	30.61	22.44	25.05	6.48	9.42	5.99	100

For each photo and screenshot that participants accessed on their phones, participants were asked: “Where is the information needed to accomplish your goal stored?” and answered by indicating their rating on a Likert-type scale that ranged from 1 (*entirely in the photo*) to 7 (*entirely in my organic memory*). Many participants chose to not answer these questions, and advanced through the question without giving a rating; however, 168 participants gave a rating for each item. These were analyzed with a paired *t*-test and the results support the general idea that offloading may be more likely with screenshotting than photographing. We found that participants’ ratings indicated that they believed that the information was more likely to be found in the image for the screenshots (*M* = 3.5, *SD* = 1.48), versus the photos (*M* = 3.83, *SD* = 1.24), *t*(167) = 2.76, *p* < 0.001, *SE*_*diff*_ = 0.08, Cohen’s *d* = 0.21.

## General discussion

The photo-taking impairment effect represents a paradox. We commonly photograph events we most want to remember, such as weddings and vacations, yet this action results in poorer memory for the photographed events. The present research extends this finding to screenshots, which are frequently taken on phones. An intuitive explanation for this effect is that the actions required to take a photo consume attentional resources that would otherwise be devoted to experiencing the event in the moment. However, our results strongly challenge the viability of this explanation, as in Experiment 2, screenshotting produced a larger impairment than taking photos with an unfamiliar tablet camera. This finding is difficult to reconcile with a divided attention account of the photo impairment effect, which would predict that the impairment would be reduced if the saving task was less complex. Similarly, the effect was comparable in magnitude when photos were taken using a familiar device (the participant’s own phone) and an unfamiliar, experimenter-provided tablet (Studies 1 and 2).

A limitation of our study is that we did not include an objective measure to establish that taking photos with an experimenter-provided tablet is more difficult than screenshotting on a computer or phone, or that photo-taking with an experimenter-provided tablet is more difficult than using one’s own phone. We based our assumptions of task difficulty on our own experiences in completing the tasks, informed by monitoring participants during the experiments and observing their ease in completing the saving tasks. To address this limitation, we later asked a separate group of participants (*n* = 24) to perform the different capturing tasks used in Studies 1–3 and rank each task in terms of difficulty, effort, and complexity. The results supported our assumptions, with participants ranking viewing as the easiest task, screenshotting as the next easiest, taking photographs with their own phone as the third easiest, and taking a photograph with the experimenter-provided tablet as the most difficult. (The difference in perceived difficulty between screenshotting and taking photos with one’s own phone was not significant, however). The details of this study and its results are available on the OSF page for this research.

This limitation notwithstanding, we believe that the current results suggest that divided attention at encoding is not an adequate explanation for the photo impairment effect. The results are, however, compatible with an account based on attentional disengagement (Soares & Storm, 2018), which posits that capturing an experience on a device triggers a broader disengagement from the experience as a whole. In this account, it is not that attention is divided between two tasks, but rather that photographing or screenshotting cues a person to be less engaged in the experience overall. Such an explanation does not rely on the secondary task being difficult or complex. One challenge to this account is that past research in naturalistic settings (e.g., restaurants and concerts) has shown that photographing experiences can actually increase engagement (Diehl et al., 2016). This discrepancy highlights the need for future research to explore the conditions under which photographing enhances versus diminishes engagement, potentially revealing important contextual or methodological factors that modulate the effects.

It is also possible to reconcile the present results with an offloading account, though with some caveats. As mentioned in the *Introduction*, the underlying mechanisms of offloading are not well specified and may overlap with attentional disengagement. However, offloading would presumably involve directing fewer cognitive resources toward encoding and maintaining information in memory when a person knows the experience has been stored externally. An offloading mechanism aligns well with the finding of exceptionally poor memory for screenshotted images, which was significantly worse than memory for photographed images in Experiment 2. The screenshot-induced impairment may be due to the fact that screenshots are often taken with the intention to offload information, as shown by survey responses in Study 4, where offloading was cited as a more common goal for screenshots than for photographs. The greater impairment in memory for screenshotted information could also reflect the fact that screenshots save an exact copy of the information, compared to the somewhat distorted image produced by photographing a computer screen. Consequently, participants may have been more likely to offload the information contained in a screenshot.

On the other hand, a key aspect of offloading is the belief that one could access the information later, and device reliability has been found to influence the likelihood of offloading (Storm & Stone, 2015). However, in our study, the impairment was comparable regardless of whether participants used their own phone (where images could be accessed later) or an experimenter-provided camera (where participants had no expectation of later access to the images). This finding seems inconsistent with a conscious offloading process.

An additional argument against an offloading account is that the memory deficit persisted despite incidental encoding instructions. Participants were not told their memory for the images would be tested, partly to simulate real-world conditions. (Although, as the results of Study 4 demonstrate, people often take photos and screenshots with memory-related goals, even though they likely do not anticipate an explicit memory test.) Another reason for using incidental encoding instructions was to compare our results with past research that used similar stimuli and procedures. Previous studies on this topic have used diverse materials and settings to demonstrate the effect, yielding inconsistent findings (e.g., Bartsch et al. found a memory improvement for photographed experiences – a result that, as far as we know, is unique). Investigating the divided attention mechanism within a single paradigm known to produce robust effects (Lurie & Westerman, 2021) seemed essential before generalizing to other paradigms (e.g., real-world settings like museums). Additionally, incidental encoding instructions allowed us to assess whether the effect stemmed from divided attention at encoding, as informing participants of a memory test might prompt them to allocate additional encoding resources to the artwork.

Incidental encoding procedures, however, may not be ideal for investigating the role of conscious offloading, as participants may feel less compelled to use the device to save information if they do not expect a memory test. Our results suggest that if offloading is occurring, it is happening at an unconscious or unstrategic level. Given how mediated our experiences are by technology, it seems plausible that participants implicitly associate capturing information digitally with later access. In this sense, some automatic, unconscious offloading may occur, reflecting a strong association between digital captures and information storage. This automatic offloading could even serve as a potential catalyst for an attentional disengagement mechanism. Further research is needed to elucidate the underlying mechanisms of the photo-taking memory impairment and to test the offloading and disengagement hypotheses more directly.

In sum, our results show that the photo-taking impairment effect is not mitigated by reducing the difficulty or complexity of the saving task. Taking photos and screenshots leads to worse memory for the saved information, and this impairment occurs whether participants use their own device to save the information or use a device provided by the experimenters. Those taking screenshots or photos may want to be selective in what they choose to save to their devices, especially if they do not plan on reviewing these images. While some argue that the use of technology may change but does not necessarily harm cognition (Cecutti et al., 2021), we note that the impairment in memory for screenshotted information is similar in magnitude to the deficit in recognition memory for amnesic patients compared to controls (Graf et al., 1984), suggesting that the use of technology to store information does create a form of “digital amnesia.”

## Data Availability

Materials are available upon request to the corresponding author. The data and data analysis files have been made available on the Open Science Framework and are accessible using the following link: https://osf.io/37anc/?view_only=1b97f5fbb3014f05b356ab76361b1f04.

## Code availability

The experiments were programmed and presented using PsychoPy (Peirce, 2007) and Pavlovia (pavlovia.org). Program files are available upon request to the corresponding author.

## Declarations

### Open science and transparency

The data and data analysis files have been made available on the Open Science Framework and are accessible using the following link:

https://osf.io/37anc/?view_only=1b97f5fbb3014f05b356ab76361b1f04.

The design and analyses of Studies 1 and 3 were preregistered. There is a preregistration available for Study 1 at https://aspredicted.org/Y32_6LD and for Study 3 at https://aspredicted.org/Q43_7PR.
